# A Case Report of Multiple Myeloma Associated With Myeloperoxidase and Proteinase-3 Antibodies Posing a Diagnostic Dilemma

**DOI:** 10.1177/2324709619843944

**Published:** 2019-05-01

**Authors:** Jordana Cheta, Michael Binder

**Affiliations:** 1 Eastern Virginia Medical School, Norfolk, VA, USA

**Keywords:** AAV and MM, multiple myeloma and ANCA vasculitis, multiple myeloma, PR3 and MPO, PR3 and MPO in multiple myeloma

## Abstract

Multiple myeloma (MM) is a clonal proliferation of antibody-producing plasma cells that can precipitate renal injury through multiple mechanisms. Antineutrophil cytoplasmic antibody (ANCA)-associated vasculitis (AAV) is an inflammatory condition that can result in renal failure through mononuclear cell infiltration and consequent destruction of glomeruli. Several case reports have identified clinical situations where differentiating these entities has been challenging. Renal biopsy is an invaluable tool in differentiating between MM and AAV when clinical uncertainty exists. We report the case of an 85-year-old man who presented with a rapid decline in renal function and serologies positive for both MM and AAV. Renal biopsy findings confirmed the diagnosis of myeloma kidney and excluded vasculitis. This case highlights an unusual clinical scenario in which both proteinase-3 (PR-3) and myeloperoxidase (MPO) antibodies are positive. While these antibodies are both individually associated with ANCA vasculitis, they are seldom simultaneously positive. Our case would suggest that positive PR-3 and MPO antibodies should raise concern for an alternative diagnosis. Indeed, ANCA, PR-3, and MPO antibodies can all be positive in patients with monoclonal gammopathy in the absence of vasculitis. Our case underscores the value of renal biopsy in the setting of MM.

## Introduction

Multiple myeloma (MM) is a clonal proliferation of plasma cells that accounts for around 10% of all hematologic malignancies.^1-3^ Knudsen et al studied renal function in 1353 patients with new-onset MM and found that 31% to 49% of patients had renal failure at the time of diagnosis.^4^ Mechanisms of renal injury in the setting of MM include dehydration, hypercalcemia, tubular involvement from cast nephropathy, amyloidosis, cryoglobulinemia, and overt glomerulonephritis from light chain deposition.^4-6^

Antineutrophil cytoplasmic antibody (ANCA)–associated vasculitis (AAV) causes renal injury due to mononuclear cell infiltrate and destruction of the vessel wall. Due to the kidneys being highly vascularized organs, vasculitis syndromes commonly affect them. AAV is described as pauci immune because it is associated with few or no immune deposits. ANCAs are autoantibodies targeted against antigens present in the cytoplasm of neutrophils and monocytes. The most common targeted antigen for ANCAs are proteinase-3 (PR-3) and myeloperoxidase (MPO). ANCA-positive patients usually have either; the occurrence of both in an individual is rare and may be due to causes that need to be further investigated, such as infections, drugs, and malignancies. ANCA is a diagnosis tool for AAV. Its presence is detected by indirect immunofluorescence (IF) and capture enzyme-linked immunosorbent assay (ELISA) methods.

Multiple myeloma has been associated with differing forms of vasculitis including ANCA-negative pauci-immune crescentic glomerulonephritis, microscopic polyangiitis with MPO-positive ANCA, and Henoch-Schonlein purpura (immunoglobulin [Ig] A-mediated vasculitis).^7-11^ In patients with hematologic malignancies, MPO and PR-3 antibodies are not reliably indicative of vasculitis.^12^ Potential mechanisms for PR-3 and MPO positivity in the absence of vasculitis include monoclonal antibody reactivity with granulocytes and/or monoclonal protein dysregulation of complement.^8,9^ Further research is needed to elucidate this interaction.

Literature review demonstrates 4 cases of AAV in the setting of MM. Three of the 4 cases had biopsy-confirmed vasculitis in the absence of the PR-3 or MPO antibodies typically associated with ANCA vasculitis.^7,13^ The fourth case had positive MPO antibodies with biopsy-proven vasculitis.^9^ In this article, we discuss an 85-year-old Caucasian male who presented with acute renal failure, monoclonal IgG kappa protein, and positive MPO and PR-3 serologies, and the importance of differentiating the mechanism of renal failure, which in turn would have significant implications on therapy (bortezomib for myeloma kidney vs cyclophosphamide and/or rituximab for vasculitis).

## Case Report

An 85-year-old Caucasian male presented with 2 to 3 months of weight loss and progressive fatigue. Past medical history was notable for hypertension, hyperlipidemia, and chronic kidney disease (stage III with baseline Cr 1.6). Home medications included amlodipine 10 mg daily and chlorthalidone 25 mg daily.

Vital signs were blood pressure 162/63 mm Hg, pulse rate 64 beats per minute, respiratory rate 14 breaths per minute, and temperature 98°F. Physical examination was remarkable for mucosal pallor.

Laboratory studies were notable for anemia with a hemoglobin of 6.6 mg/dL, acute renal failure with a serum creatinine of 10.1 mg/dL, positive antinuclear antibody, positive MPO, positive PR-3, and positive ribosomal antibodies. Serum ANCA was negative. Urinalysis was notable for proteinuria (3+) with red blood cells. The 24-hour urine protein was 2416 mg. Kappa to lambda light chain ratio was elevated at 108.3. Serum protein electrophoresis was significant for an elevated monoclonal IgG protein of 4676 mg/dL (reference range = 700-1600 mg/dL) with kappa light chains (see Table 1).

**Table 1.** Laboratory values.

Parameter	Results	Reference Range
Hemoglobin	6.6	13.5-17.4 g/dL
Creatinine	10.1	<1.2 mg/dL
24-hour urine protein	2416	0-99 mg/24 hours
Antinuclear antibody	Positive titer	Negative
Double-stranded DNA antibody	Negative	Negative
Anti-smith antibodies	Negative	Negative
Anti-Jo-1 antibodies	Negative	Negative
Anti-ribosomal protein	1.7	0.0-0.9 AI
Anti-scleroderma antibodies	Negative	Negative
Sjogren-SSA, Sjogren-SSB antibodies	Negative	Negative
P-ANCA and C-ANCA	Negative	Negative
MPO antibody	16.2	0.0-0.9 U/mL
PR-3 antibody	15.9	0.0-3.5 U/mL
Complement, C3 and C4	C3 = 146, C4 = 17	C3: 82-167 and C4: 14-44 mg/dL
Hepatitis B Ag and C antibody	Negative	Negative
Cryoglobulin	None detected	None detected

**Figure 1.**
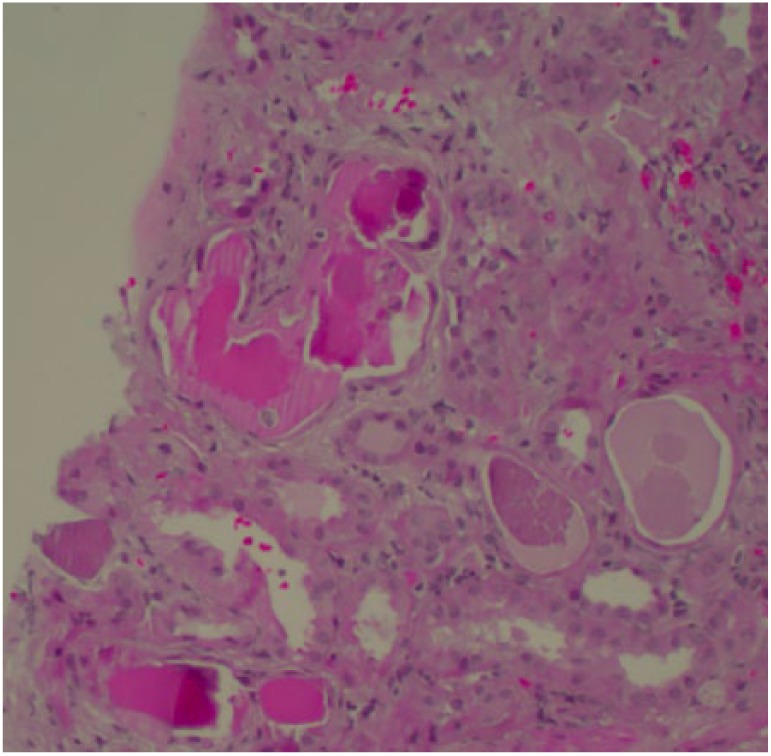
Kidney biopsy under light microscopy with periodic acid-Schiff (PAS) staining. Diffuse acute tubular injury of tubules. Atypical casts stain pink with a granular to fractured appearance. Note the jagged edges with a surrounding cellular reaction.

Given the acute renal failure with hematuria, proteinuria, and laboratories suggestive of MM and AAV, a renal biopsy was warranted to confirm a diagnosis. The biopsy assessed 23 glomeruli, none sclerotic. The biopsy was significant for mild mesangial expansion, diffuse acute tubular injury, and atypical casts with a granular to fractured appearance with a surrounding cellular reaction (Figure 1). IF demonstrated no glomerular or extraglomerular staining with IgG, IgA, IgM, C3, C1q, fibrin, or lambda light chains. The intratubular atypical casts demonstrated strong monoclonal staining with kappa light chains. On electron microscopy, the glomeruli were unremarkable with intact foot processes on the basement membranes. No immune complex dense depositions or fibrillar deposits were identified.

Given clinical concern for MM, a bone marrow biopsy was performed. It demonstrated atypical plasmacytosis consistent with MM. Plasma cells represented 65% of the marrow. The patient’s MM was treated with bortezomib and 5 sessions of 1.0 volume plasma exchange with albumin. Hemodialysis was initiated for rapidly worsening renal failure. Unfortunately, there was no significant renal recovery, and the patient remained hemodialysis-dependent.

## Discussion

Kidney disease in the setting of MM is common and usually associated with immune deposits and cast nephropathy. The causes of renal insufficiency vary in patients with MM. Prior biopsy reviews of 118 patients with MM by Montseny et al have revealed myeloma kidney in 48% of patients, AL amyloidosis in 30%, light chain deposit disease in 19%, chronic tubulointerstitial nephritis in 10%, and cryoglobulins in <1%.^14^ The positive PR-3 and MPO antibodies present in our case raised concern for vasculitis as an alternative etiology for renal dysfunction. Case reports suggest a possible association between MPO-ANCA and ANCA-negative vasculitis with MM. Esnault et al studied serum from 125 patients with MM with IF and radioimmunoassay and found 7 with positive IgG-ANCA and 5 with positive IgM-ANCA.^5,7,9,15^ Given the differing treatments between MM and systemic vasculitis, a renal biopsy was necessary for definitive diagnosis. The renal biopsy revealed glomeruli with mesangial expansion and atypical casts, which stained positive for monoclonal kappa light chains. These results suggest the presence of myeloma kidney with cast formation caused by the precipitation of excess light chains with Tamm-Horsfall protein.^16^ Based on the results of the kidney biopsy, therapy for MM was initiated.

ANCAs can be present in a variety of different hematologic and connective tissue disorders without a vasculitis being present. Ruffatti et al studied 115 patients with systemic sclerosis and tested using indirect IF and ELISA and found 2 patients with indirect IF positivity along with MPO and PR-3 ELISA positivity and 3 patients with IF-negative but ELISA-positive results.^17^ In addition, studies have shown ANCA associated with systemic lupus erythematosus, rheumatoid arthritis, polymyositis, Sjogren syndrome, and others, although the specificity of IF and ELISA testing for vasculitis in patients with connective tissue disease was still found to be 99.5%.^18^ In 2 studies of hematologic malignancies, positive ANCAs were found without evidence of vasculitis in 8 out of 60 patients with Hodgkin’s lymphoma and 6 patients out of 140 with lymphoid malignancies.^12,19^

There have been 4 previous case reports of AAV coexisting with MM (Table 2). Our case is unique in that the patient’s renal failure was due to MM and not AAV despite positive MPO and PR-3 ANCA. Anaele et al reported a 57-year-old woman who presented with end-stage renal failure due to AAV with negative serologies in the setting of MM who was treated with bortezomib. She had resolution of her MM but remained hemodialysis-dependent.^7^ Grundmann et al reported a 60-year-old male with MM who presented with rapidly progressive renal failure due to AAV with negative ANCA serologies.^13^ He initially failed therapy with cyclophosphamide, steroids, rituximab, cyclosporine, and azathioprine but then had resolution of his MM and renal dysfunction function with bortezomib.^12^ Rope et al reported a 58-year-old man with MM who presented with renal failure and arthritis due to AAV with negative serologies. He was treated with cyclophosphamide and bortezomib with resolution of his MM and improvement in renal function.^8^ In the first reported case of MM with AAV, Kapoulas et al described a 72-year-old male with MM (monoclonal IgG lambda as opposed to the other case reports, which were all IgG kappa) who presented with renal failure due to AAV with positive MPO antibodies (vs negativity for all the previous case reports and MPO and PR-3 positivity for our case). The patient was treated with 7 cycles of plasma exchange, cyclophosphamide, and steroids with recovery of renal function. Unfortunately, the patient experienced fatal pulmonary hemorrhage from vasculitis.^9^ These cases illustrate several important concepts: (1) differentiating between myeloma kidney and AAV can be difficult in patients with monoclonal gammopathy, (2) renal biopsy should be performed to establish a diagnosis in patients with concomitant concern for AAV and myeloma kidney, and (3) AAV cases may have negative serologies if indirect IF is utilized instead of ELISA.

**Table 2.** Comparison With Previous Case Reports of MM and AAV and Their Outcomes.

Case	Age/Sex	Clinical Presentation	Antibody Serology	Renal Biopsy Results	Therapy	Treatment Outcome
Current case (2018)	85/male	AKI, weight loss, anemia	ANCA− MPO+ PR-3+ monoclonal kappa IgG	Intratubular atypical casts with monoclonal kappa light chains	1. Velcade 2. Plasma exchange (5 treatments)	Hemodialysis dependent
Rope et al^8^	58/female	AKI, arthritis	ANCA− Monoclonal kappa IgG	PIGN and arteriolar vasculitis with crescents No immunoglobulin deposits	1. Velcade 2. CyC/steroids	Improvement of renal function. Died from fatal pulmonary hemorrhage from systemic anticoagulation for venous thromboembolism.
Anaele et al^7^	57/female	ESRD from MM	ANCA− Monoclonal kappa IgG	Chronic sclerosing PIGN	1. Velcade	Hemodialysis-dependent resolution of MM
Grundmann et al^13^	60/male	RPGN	ANCA− Monoclonal kappa IgG	PIGN (4/14 crescents), lymphocytic infiltration and 20% interstitial fibrosis and mild tubular atrophy. No paraprotein deposits	1. Velcade 2. CyC/steroids (failed) 3. Rituximab/CsA/Azathioprine (failed)	Resolution of renal function and MM with Velcade
Kapoulas et al^9^	72/male	AKI, weight loss, anemia	MPO+ Monoclonal lambda IgG	70% of glomeruli with focal necrosis, inflammatory cell infiltrate, and cellular crescents	1. Plasma exchange (7 treatments) 2. CyC/steroids	Renal function recovered. Died of pulmonary hemorrhage from pulmonary vasculitis.

Abbreviations: MM, multiple myeloma; AAV, antineutrophil cytoplasmic antibody (ANCA)-associated vasculitis; AKI, acute kidney injury; MPO, myeloperoxidase; PR-3, proteinase-3; IgG, immunoglobulin G; PIGN, pauci-immune glomerulonephritis; CyC, cyclophosphamide; ESRD, end-stage renal disease; RPGN, rapidly progressing glomerulonephritis; CsA, cyclosporine.

ANCA positivity is relatively rare in patients with hematologic malignancies and does not always indicate the presence of vasculitis. In a study by Rao et al, 2345 samples of serum over 2 years were tested for ANCAs by indirect IF and by ELISA.^20^ A total of 2.1% of the samples were positive by ELISA but had a negative indirect IF. Of these cases with positive ELISA and a negative indirect IF, only 1 patient was diagnosed with an ANCA-associated vasculitis. The remaining patients had nonvasculitic inflammatory conditions such as systemic lupus erythematosus and inflammatory bowel disease.^20^ The 1999 International Consensus on ANCA testing dictates that indirect IF should be used as a screening tool and confirmed by ELISA for MPO and PR-3.^21^

## Conclusion

This case helps highlight the rarity of both a positive MPO and PR-3 antibody test, and that positive serologies with negative IF usually do not indicate vasculitis. Renal biopsy is an invaluable tool when diagnostic uncertainty exists, as therapy for vasculitis should only be initiated when signs of active vessel destruction are present on biopsy.
